# Efficacy of onabotulinumtoxinA in the treatment of unipolar major depression: Systematic review, meta-analysis and meta-regression analyses of double-blind randomised controlled trials

**DOI:** 10.1177/0269881121991827

**Published:** 2021-03-15

**Authors:** Danilo Arnone, Hassan Galadari, Carl J Rodgers, Linda Östlundh, Karim Abdel Aziz, Emmanuel Stip, Allan H Young

**Affiliations:** 1 United Arab Emirates University, College of Medicine and Health Sciences, Al Ain, United Arab Emirates; 2 Centre for Affective Disorders, Psychological Medicine, Institute of Psychiatry, Psychology and Neurosciences, King’s College London, London, UK; 3 Centre Hospitalier Universitaire de Montreal (CHUM), Institute Universitaire en Santé Mentale de Montréal, Université de Montreal, Montreal, Canada

**Keywords:** OnabotulinumtoxinA, double-blind randomised controlled trial, major depression, meta-analysis

## Abstract

**Background:**

OnabotulinumtoxinA is a novel therapeutic intervention whose mechanism of action is believed to modify the negative facial feedback, thus abating symptoms of depression. This putative new antidepressant agent offers minimal systemic side effects and negligible risk of pharmacological interactions. We set out to examine the evidence for the use of onabotulinumtoxinA in major depression.

**Methods:**

A systematic search of the literature identified double-blind randomised controlled trials (RCTs) investigating the use of onabotulinumtoxinA in the treatment of major depression versus placebo. Data, reported according to the Preferred Reporting Items for Systematic reviews and Meta-Analyses (PRISMA), was combined in meta-analyses (PROSPERO registration ID: CRD42020183538).

**Results:**

The search identified five RCTs (four double-blind) comparing onabotulinumtoxinA to placebo. OnabotulinumtoxinA was more effective than placebo when administered within the 20–40 IU dose range in double-blind RCTs. The analysis was free of publication bias and significantly heterogeneous. Meta-regression analyses indicated that onabotulinumtoxinA was more efficacious in women and in higher doses in female patients and less effective with polypharmacy, especially when an increasing number of antidepressants were prescribed. The effectiveness of onabotulinumtoxinA was higher in more recently published double-blind RCTs.

**Conclusion:**

The meta-analysis supports the efficacy of the intervention with the results being highly heterogeneous across studies. In view of the heterogeneity of the findings and the significant moderators of benefit (sex, year of study completion and the interaction between sex and dose), more research is required to better understand the role of onabotulinumtoxinA in the treatment of depression.

## Introduction

Major depression is a common condition worldwide, reaching a life prevalence of 15% in high-income countries (in 2002). Up to 65% of individuals treated for a depressive episode do not fully respond to interventions or achieve full remission (Cleare et al., 2015). The Sequenced Treatment Alternatives to Relieve Depression trial (STAR*D), the largest community study to date evaluating treatment response in major depression, suggested that approximately 31% of individuals tend to achieve remission after two treatment steps. Full remission becomes less likely with successive steps, contributing to the chronicity of the condition (Rush et al., 2006; Warden et al., 2007). Frequency and chronicity of the disorder are responsible for the vast societal and financial impact of major depression. This hereby deprives affected individuals of quality of life in lieu of the high number of years lived with a disability and premature death (WHO, 2002). Furthermore, especially in refractory cases, more aggressive use of pharmacology and polypharmacy increase the risk of adverse effects, with a negative impact on adherence to treatment and ultimately on clinical outcome (Cleare et al., 2015). There is undoubtedly a need for novel approaches to treat major depression, especially in cases of inadequate response to antidepressant treatment. Novel therapeutic interventions with minimum side effects would be ideal for future treatment options in order to maximise adherence and minimise the occurrence of pharmacological interactions. Several studies have shown that the single injection of onabotulinumtoxinA into the facial muscles of the glabellar region is an effective, novel and well-tolerated treatment for major depression. OnabotulinumtoxinA is a neurotoxin, which induces transitory muscle paralysis by blocking the release of acetylcholine from the nerve endings. It is primarily indicated to treat conditions caused by excessive or spasmodic muscle contractions (Satriyasa, 2019). In 2002, the Food and Drug Administration approved Botox® Cosmetic (onabotulinumtoxinA) to treat moderate to severe frown lines caused by glabellar contraction (Monheit and Pickett, 2017). The putative mechanism of action postulates that facial expressions, including frowning, produce sensory feedback, which can negatively influence emotions. When injected for such an indication in individuals with major depression, onabotulinumtoxinA is believed to modify ongoing emotional responses by providing a positive cue which reduces ‘facial negative feedback’, resulting in measurable beneficial clinical response without any major side effects (Ascher et al., 2004; Coles et al., 2019). This work appraises the evidence from randomised controlled trials (RCTs) for the use of onabotulinumtoxinA in major depression. Previous work has indicated that onabotulinumtoxinA is efficacious in treating major depression (Coles et al., 2019; Parsaik et al., 2016). The aim of this meta-analysis was to review systematically all of the available data, primarily extracted from randomised, double-blind, placebo-controlled trials, on the use of onabotulinumtoxinA for the treatment of major depression.

## Methods

### Search strategy and study selection

A comprehensive literature search including six electronic databases (PubMed, EMBASE, PsycInfo, Cochrane Library, Scopus and Web of Science) was conducted from their inception to November 2020. Additionally, selected clinical trial registers and grey repositories were searched. PubMed and PubMed’s MeSH were used in the pre-search phase in order to develop the strategy to identify search-term variations systematically which were subsequently applied to all databases, including grey materials. A combination of the search fields ‘title’, ‘abstract’ and ‘MeSH’/‘thesaurus’ was used to retrieve the best possible result for all included search terms, with no filters or limitations. Key search terms included ‘randomised controlled trial’ OR ‘double-blind controlled trial’ AND ‘bipolar and related disorders’ OR ‘mood disorders’ OR ‘depression’ OR ‘affective disorders’ AND ‘botulinum toxins/botulinum toxins type A/Clostridium botulinum A toxin/Botox/onabotulinumtoxinA/botulinum neurotoxin type-A’ (see Supplemental Material 1). All records were uploaded to the systematic review software Covidence (Veritas Health Innovation, Melbourne, Australia) for automatic de-duplication and blinded screening. Identified papers meeting the inclusion criteria were extracted and cross-referenced. The Preferred Reporting Items for Systematic reviews and Meta-Analyses (PRISMA) was adopted for the selection process combined in a PRISMA flow diagram (Moher et al., 2009; PROSPERO registration ID: CRD42020183538).

### Eligibility criteria

The searches aimed to identify RCTs that evaluated the efficacy of onabotulinumtoxinA in comparison to placebo in major depression. Within these trials, preference was given to double-blind RCTs. Included studies assessed depression according to standardised diagnostic criteria and established severity according to validated depression rating scales. Studies had to evaluate response to onabotulinumtoxinA in monotherapy or in addition to a stable regimen of antidepressant drugs at specific time points. Patients with any significant pathology other than unipolar major depression (e.g. DSM IV Axis I pathology other than secondary stable anxiety disorders) were excluded. Other exclusion criteria were: current alcohol/substance misuse, a diagnosis of personality disorder or any use of onabotulinumtoxinA to treat conditions other than major depression or for cosmetic indications.

### Data extraction, methodological quality appraisal and outcome measures

Two independent assessors (D.A. and C.R.) reviewed all the literature. A third author (K.A.A.) resolved conflicts by consensus. Quality assessment of the selected manuscripts was conducted by using the Revised Cochrane risk-of-bias tool for randomised trials (Sterne et al., 2019). The main outcome measure was the mean change in depression rating scale score in the group treated with onabotulinumtoxinA in comparison to placebo at the study end point.

### Data synthesis and analysis

A random effect meta-analysis was conducted with STATA v9.0 (Stata Corp, College Station, TX) supplemented by ‘Metan’ software downloadable from the Centre for Statistics in Medicine (Oxford, UK), as previously described (Arnone et al., 2009, 2012, 2018). In brief, we used Cohen’s *d* to calculate standardised mean differences in remission and response rates of onabotulinumtoxinA in comparison to placebo, calculated as mean and standard deviation. The *Q*-test evaluated the presence of heterogeneity. If the *Q*-test was significant, Galbraith plot served to identify studies contributing to heterogeneity. The proportion of effect size attributable to heterogeneity was calculated with *I*^2^ (Higgins et al., 2003). As many confounders as possible were considered to explain heterogeneity. Clinical and demographic variables which were available for consideration included: year of publication, age, sex, age of onset, duration of the depressive episode, severity of depression, presence and number of adjunctive pharmacological treatments, number of units of onabotulinumtoxinA injected, severity of frown lines prior to treatment, adoption of blinding and intention to treat methodologies to minimise the occurrence of bias. Egger’s test was used to evaluate the occurrence of publication bias, with the significance level set at *p* < 0.05 (Egger et al., 1997). Rating scales scores were converted to Montgomery–Åsberg Depression Rating Scale (MÅDRS) scores by using established conversion formulae (Hawley et al., 1998).

## Results

### Study selection, description of the studies and quality assessment

**Figure 1.**
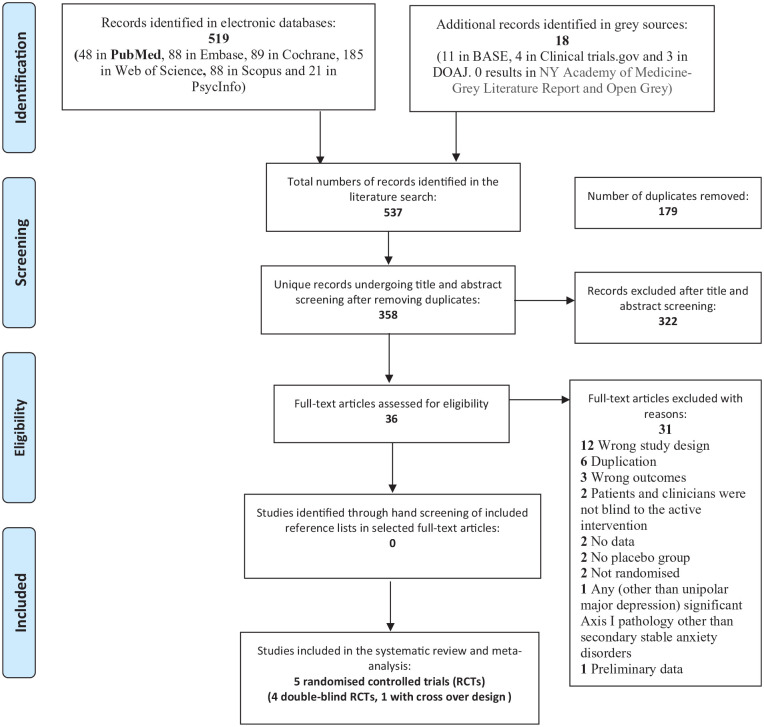
Preferred Reporting Items for Systematic reviews and Meta-Analyses (PRISMA) flow chart.

The search identified 537 papers, of which 358 remained after de-duplication. Five RCTs comparing onabotulinumtoxinA to placebo were identified (see Figure 1). Table 1 shows that the studies treated 230 patients with onabotulinumtoxinA, primarily women (91%), with a mean age of 46 years, a mean disease duration of 12 years and a mean MÅDRS severity score at study inclusion of 27. It was possible to combine the studies only at week 6 when the mean reduction on MÅDRS score, following the active intervention, was around 47%. No other time points were sufficiently similar across the studies to allow data combination. Mean doses of onabotulinumtoxinA were 26 IU in men and 33 IU in women. Four of the five studies met the full eligibility criteria, as shown in the PRISMA flow diagram (Figure 1; Brin et al., 2020; Finzi and Rosenthal, 2014; Magid et al., 2014; Wollmer et al., 2012). With regard to the dose of onabotulinumtoxinA, the trials utilised doses similar to those used to treat glabellar frown lines for cosmetic indications (20–40 IU; Carruthers and Carruthers, 1992; Carruthers et al., 1996; Satriyasa, 2019). The most recent and largest study (56% of total number of participants) by Brin et al. evaluated two groups of women treated with two doses of onabotulinumtoxinA (30 and 50 IU) in monotherapy. Both groups were included in the analyses. This was the only study which used onabotulinumtoxinA in monotherapy. The dose of 50 IU was chosen to paralyse the functionality of facial muscles of the glabellar region completely beyond the 20–40 IU commonly used in cosmetic procedures (Brin et al., 2020; Carruthers and Carruthers, 1992; Carruthers et al., 1996). Magid et al. (2014) involved two groups, both included in the analyses, that were randomised to the active compound and placebo at different time points by using a crossover design. A fifth randomised controlled trial by Zamanian et al. (2017) did not meet the full criteria for a double-blind RCT, as it did not adopt any blinding strategy.

**Table 1.** Studies included in the meta-analysis.

Author	Year	*N*	Age (years)	Males (*N*)	Females (*N*)	Rating scale	MÅDRS (C) severity	Disease duration (years)	Criteria	Time to response	Units males (*N*)	Units females (*N*)	% MÅDRS (C) reduction
Wollmer et al.	2012	15	52.2	3	12	HAMD17	21.4	11.97	DSM IV	6 weeks	39	29	48.3
Finzi et al.	2014	41	47.9	9	32	MADRS	31.6	–	DSM IV	6 weeks	40	29	46.5
Magid et al., I	2014	11	48.5	0	11	HAMD21	22	16.09	DSM IV	6 weeks	39	29	58.7
Magid et al., II	2014	19	50	2	17	HAMD21	20.16	18.05	DSM IV	6 weeks	39	29	43.2
Zamanian et al.	2017	14	35.14	7	7	BDI	30.86	3.71	DSM 5	6 weeks	–	–	43.4
Brin et al. (30 IU)	2020	65	43.6	0	65	MÅDRS	32	12.3	DSM IV-TR	6 weeks	0	30	36.2
Brin et al. (50 IU)	2020	65	44.4	0	65	MÅDRS	32	12.2	DSM IV-TR	6 weeks	0	50	35.9
*Mean values (%)*		*230*	*45.97*	*21 (0.091%)*	*209 (90.8%)*		*27.14*	*12.39*			*26.16*	*32.67*	*44.6*

HAMD17: Hamilton Depression Rating Scale 17 items; HAMD21: Hamilton Depression Rating Scale 21 items; BDI: Beck Depression Rating Scale; MÅDRS: Montgomery-Åsberg Depression Rating Scale; MÅDRS (C): Montgomery-Åsberg Depression Rating Scale converted scores.

Assessment of quality suggested that the four randomised, double-blind, placebo-controlled studies were well designed and overall at low risk of bias aside performance bias (blinding of participants and personnel) and detection bias (outcome assessment).

The main source of bias across these studies was the difficulty in blinding participants in view of the physical effects of onabotulinumtoxinA on the face. These effects are necessary for the efficacy in the treatment of depression. The other risk was related to study assessors correctly guessing the intervention delivered, irrespective of masking strategies. The study by Zamanian et al. (2017), which was randomised but not double-blind, was of lower quality and had a high risk of bias on all domains of the quality assessment tool.

### Meta-analyses

We present below the results of the double-blind RCTs which met the full inclusion criteria and used onabotulinumtoxinA within the recommended 20–40 IU (Brin et al., 2020; Finzi and Rosenthal, 2014; Magid et al., 2014; Wollmer et al., 2012). Then, we present analyses of the group treated with 50 IU onabotulinumtoxinA (Brin et al., 2020) and of all the RCTs, including the study by Zamanian et al. (2017) which did not use blinding techniques.

#### Meta-analysis of double-blind RCTs (20–40 IU onabotulinumtoxinA)

**Figure 2.**
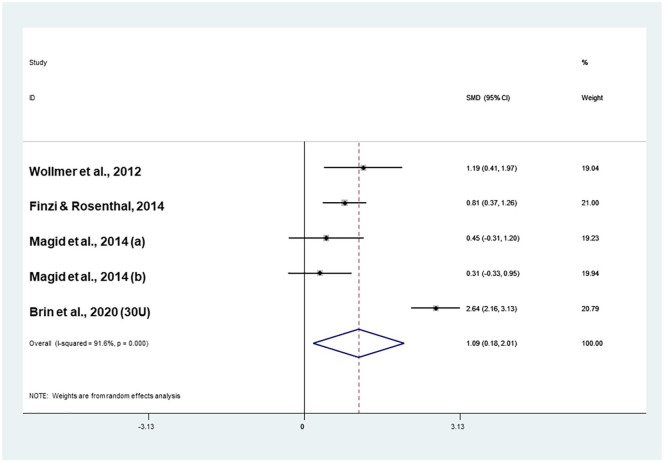
Meta-analysis of four double-blind randomised controlled trials (RCTs) with 20–40 IU onabotulinumtoxinA.

When onabotulinumtoxinA was tested in double-blind RCTs and administered within the 20–40 IU range, it was more effective compared to placebo (effect size = 1.09; confidence interval (CI) 0.18–2.01) in the absence of publication bias (Coef.: 5.1; *p* = 0.54; Figure 2). This analysis was highly heterogeneous (*I*^2^ = 91.6%; *p* < 0.001). Exploration of heterogeneity suggested that onabotulinumtoxinA was more effective compared to placebo in more recently published studies (Coef.: 0.26; *Z* = 2.82; *p* = 0.005), in female patients (Coef.: 0.04; *Z* = 3.54; *p* < 0.001) and in women who received higher doses (Coef.: 1.94; *Z* = 6.66; *p* < 0.001). Conversely, onabotulinumtoxinA was less effective if administered in polypharmacy (Coef.: −1.94; *Z* = −6.66; *p* < 0.001) and with an increasing number of antidepressants (Coef.: -1.26; *Z* = -2.68; *p* = 0.007; Table 2).

**Table 2.** Meta-regression analyses.

	Year	Age	Males	Females (*N*)	ADTs+	ADTs (*N*)	Age onset	Duration illness	Episode duration	Illness severity	Com+	Double blind+	Intention-to-treat analysis	BTX units males	BTX units (females)	Frown line severity (baseline)
*All studies combined*																
Coefficient	−0.01	−0.02	0.032	−0.0005	−0.13	−0.14	−0.13	−0.042	−0.006	0.01	0.23	−0.23	0.038	−0.002	−0.1	−0.14
*Z*-value	−0.06	−0.18	0.22	−0.03	−0.01	−0.16	−0.19	−0.33	−0.02	0.05	0.16	−0.16	0.04	−0.1	−2.04	−0.91
*p*-Value	0.95	0.86	0.82	0.98	0.99	0.88	0.85	0.74	0.98	0.96	0.87	0.87	0.97	0.92	0.042*	0.36
*Double-blind RCTs only*																
Coefficient	−0.01	−0.013	0.026	0.001	−0.036	−0.14	−0.13	−0.1	−0.001	0.04	NA	NA	0.11	−0.001	−0.1	−0.14
*Z*-value	−0.07	−0.07	0.15	0.04	−0.03	−0.16	−0.19	−0.33	−0.02	0.14	NA	NA	0.09	−0.03	−2.04	−0.91
*p*-Value	0.95	0.95	0.88	0.97	0.98	0.88	0.85	0.74	0.98	0.89	NA	NA	0.92	0.98	0.042*	0.36
*Double-blind RCTs, 20–40* *IU dose range*																
Coefficient	0.26	−0.22	−0.07	0.04	−1.94	−1.26	NA	−0.29	−0.02	0.29	NA	NA	1.08	−0.00	1.94	−0.14
*Z*-value	2.82	−1.85	−0.49	3.54	−6.66	−2.68	NA	−1.83	−1.20	1.53	NA	NA	1.37	−0.06	6.66	−0.91
*p*-Value	0.005*	0.064	0.62	0.000*	0.000*	0.007*	NA	0.067	0.23	0.127	NA	NA	0.17	0.95	<0.001*	0.36

* Meta-regressions which reached statistical significance.

ADT+: effect of co-administered antidepressants; ADTs (*N*): number of antidepressants administered; BTX: onabotulinumtoxinA; Com+: presence of co-morbidity; RCT: randomised controlled trial; NA: not available.

#### Meta-analysis of double-blind RCTs

**Figure 3.**
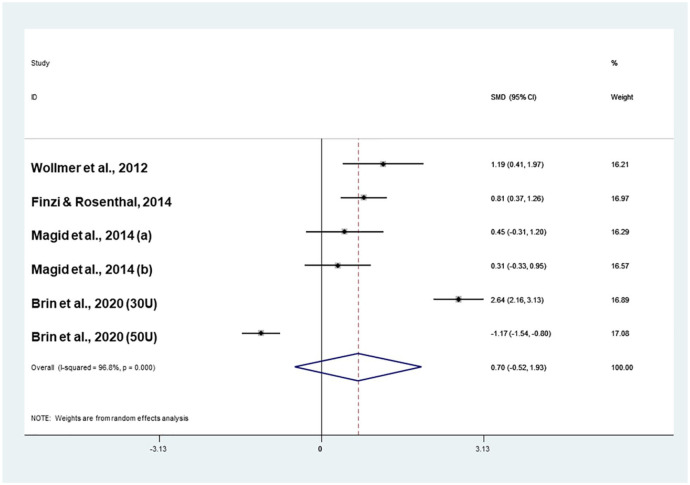
Meta-analysis of four double-blind RCTs including all comparisons (20–40 IU and 50 IU onabotulinumtoxinA).

When the group treated with 50 IU onabotulinumtoxinA was included in the analysis (Brin et al., 2020), onabotulinumtoxinA was not more effective than placebo (effect size: 0.70; CI −0.52 to 1.93) in the absence of publication bias (Coef.: 7.3; *p* = 0.46; Figure 3). The analysis was highly heterogeneous (*I*^2^ = 96.8%, *p* < 0.001). Systematic exploration of heterogeneity suggested that the efficacy of onabotulinumtoxinA in comparison to placebo decreased when the dose of toxin administered to women increased (Coef: −0.1; *Z* = −2.04; *p* = 0.042; Table 2).

#### Meta-analysis of the five RCTs

**Figure 4.**
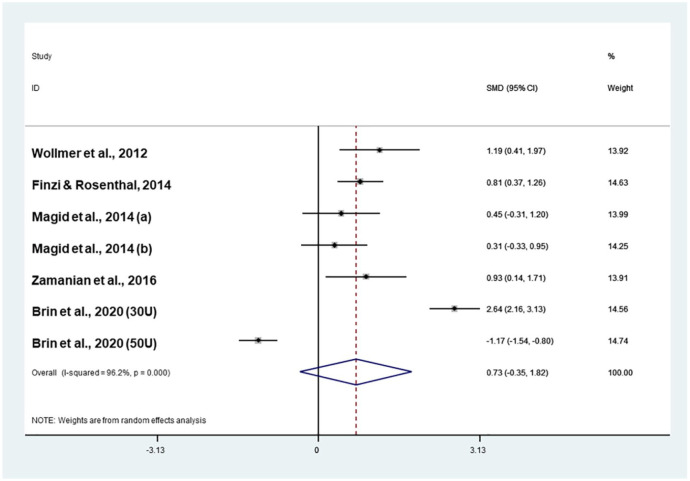
Meta-analysis of five RCTs.

When the study by Zamanian et al. (2017) was included in the analysis, onabotulinumtoxinA was not more effective than placebo (effect size: 0.73; CI −0.35 to 1.82) in the absence of publication bias (Coef.: 6.37; *p* = 0.4; Figure 4). The analysis was highly heterogeneous (*I*^2^ = 96.2%, *p* < 0.001). Systematic exploration of heterogeneity suggested that the efficacy of onabotulinumtoxinA in comparison to placebo decreased with an increasing dose of the toxin in women (Coef.: −0.1; *Z* = −2.04; *p* = 0.042; Table 2).

## Discussion

In this work, we set out to evaluate the efficacy of onabotulinumtoxinA in the treatment of major depressive disorders in comparison to placebo through a systematic appraisal of data published from double-blind RCTs.

Three meta-analyses tested the superiority of onabotulinumtoxinA in comparison to placebo by estimating the summary effect size of differences in depression scores of onabotulinumtoxinA and placebo in: (a) double-blind RCTs that administered 20–40 IU onabotulinumtoxinA, (b) double-blind RCTs including a dose of 50 IU onabotulinumtoxinA and (c) all the available RCTs. OnabotulinumtoxinA was superior to placebo only when administered within the 20–40 IU dose range.

Our work expands on two previous meta-analyses by Coles et al. (2019) and Parsaik et al. (2016). Parsaik et al. included the three small RCTs available at the time and reported that the mean difference in depression scores between onabotulinumtoxinA and placebo was as large as -9.80 points difference of rating scales (95% CI -12.90 to -6.69). A more recent meta-analysis by Coles et al., which included four of the five RCTs (three double-blind), concluded that onabotulinumtoxinA was superior to placebo in reducing depression scores after six weeks of treatment with an effect size of 0.83 (95% CI 0.52–1.14). The meta-analysis presented here adds to the work of Coles et al. by including the largest study to date recently published in 2020 sponsored by Allergan. This trial by Brin et al. (2020) comprises 56% of the all the patients treated with onabotulinumtoxinA for a major depressive episode. Our results support the superiority of onabotulinumtoxinA to placebo, with an effect size of 1.09 (95% CI 0.18–2.01), but only when used within the 20–40 IU range. Meta-regression analyses suggested that more recently published studies contributed more to the efficacy of onabotulinumtoxinA, likely related to the largest study by Brin et al. Similarly, meta-regressions indicated that onabotulinumtoxinA was more efficacious in women (*p* < 0.001) and in higher doses in female patients (*p* < 0.001). This is likely to be driven by Brin et al.’s (2020) study, which only included women.

As part of the systematic review, we conducted a quality assessment of the studies. The appraisal of the work included overall, suggests that the main weakness of the double-blind RCTs is the risk of bias introduced by the potential un-blinding of patients and assessors. This is because despite the precautions adopted by the researchers, participants given onabotulinumtoxinA often noted the effect of the toxin, and clinicians, even if unaware of the intervention, became aware during their interaction with the patients. In the study conducted by Wollmer et al. (2012), 90% of the participants and 60% of assessors guessed the intervention correctly. In the study by Finzi and Rosenthal (2014), 73% of the assessors, 52% in the onabotulinumtoxinA treated and 46% in the placebo group identified the intervention correctly. Magid et al. (2014) did not ascertain whether blinding had worked in their study. Brin et al. (2020) adopted remote scoring to control for assessor bias so that approximately 24% of assessors guessed correctly across the different groups. In the same study, 66% of patients and 46% of the clinicians who assessed participants in person identified the intervention correctly in the 30 IU group. In the 50 IU group, 79.2% of patients and 62.3% of assessors guessed the intervention correctly.

Meta-regression analyses suggested that within the 20–40 IU dose range, onabotulinumtoxinA was more effective in women, especially if female patients received higher doses. This is consistent with the observation that doses causing sufficient paralysis are effective, whereas a dose in the range of 50 IU causing complete paralysis of the glabellar muscles may be more likely to un-blind the intervention and modify the balance between active and placebo effects. However, this observation poses some questions regarding the contribution of the placebo effect to the overall efficacy of onabotulinumtoxinA. In fact, although un-blinding did not have a measurable bearing on the statistical results of each individual trial, quality assessment of the studies suggested that blinding techniques might have introduced a source of bias into these studies. This is not unique to onabotulinumtoxinA trials in major depression. For example, double-blind trials in the treatment of chronic migraine faced a similar issue (Diener et al., 2010).

A further complication is the notion that the reduction in the motor activity of the corrugator and procerus muscles (Carruthers and Carruthers, 1992) is the putative underlying mechanism responsible for the antidepressant action of onabotulinumtoxinA according to the ‘facial feedback hypothesis’. This hypothesis suggests that the toxin blocks negative facial expressions which feedback into a biased cognitive appraisal towards self and others responsible for low mood, resulting in mood improvement beyond the duration of action of the toxin (Coles et al., 2019). It is important to highlight that this mechanism was not tested formally in the included studies, and it is therefore difficult to conclude how this intervention works.

Based on this mechanism of action, a ‘non-interventional’ type of placebo might not be the best comparator for testing the efficacy of onabotulinumtoxinA. This is because of the difficulty in attributing the therapeutic effect due to the procedure itself or to the paralysis of the glabellar muscles. Perhaps an alternative method in future studies might be to inject onabotulinumtoxinA in another location as a way to establish whether response is related to any unrelated effects of the toxin or to the specific paralysis of the corrugator and procerus muscles consistent with the ‘facial feedback hypothesis’. However, it is difficult to think about which site would be suitable. Perhaps a viable alternative might be a comparison to ‘fillers’, which would have the same cosmetic improvement but would not reduce the ability to show negative facial expressions.

Another limitation to the current data is that the response to onabotulinumtoxinA was consistently measured at week 6 in all the studies, which is shorter than the duration of action of the toxin. Some studies reported additional outcome data at different time points after week 6. Unfortunately, the information varied extensively in relation to time points but also content and quality of the data. This prevented the possibility of combining the information in meta-analyses after week 6. Brin et al. (2020) reported that the group treated with 30 IU of onabotulinumtoxinA improved up to week 15, although the difference, in comparison to placebo, did not reach statistical significance. The authors reported, however, that patients who did not benefit from the intervention were allowed to exit the study in the case of relapse after week 12, which weakens the claim of efficacy. Similarly, Wollmer et al. (2012) reported improvement up to week 16, although patients’ treatment was not maintained constant. Magid et al. (2014) reported a statistically significant further improvement in depression scores up to 24 weeks in the nine (81%) patients who were first treated with the toxin in the crossover trial. This study included a very small number of participants and did not adopt an intention-to-treat analysis. Therefore, it is possible that patients who continued in the study were also those who benefitted from the intervention the most.

As Coles et al. (2019) noted, the effect size of onabotulinumtoxinA in comparison to placebo is large. Our work suggests an effect size of 1.09 is much larger than the effect size of ‘facial feedback effects’ in the 0.17–0.42 range and of antidepressants versus placebo trials equivalent to approximately 0.30 (Cipriani et al., 2018). This is in support of the notion that placebo effect might play a role in the studies reviewed here, particularly when a total paralysis of the muscles occurs (50 IU in the study by Brin et al., 2020). Doses in the range of 50 IU are known to abolish functionality of the corrugator muscle (Pribitkin et al., 1997), perhaps increasing patients’ expectations and placebo effects (Brin et al., 2020). However, although not specifically related to psychiatric disorders, a study comparing onabotulinumtoxinA with amitriptyline for the prophylactic treatment of chronic daily migraines suggested that the two interventions were of a similar effect size (Magalhães et al., 2010). In this meta-analysis, the estimate number needed to treat, based on an average 40% response rate in the experimental group and an effect size of 1.09, is equivalent to three to four (Furukawa, 1999).

Another observation is that in the meta-regression analyses, onabotulinumtoxinA was less effective when administered with other antidepressants, especially when the number of compounds was higher. This might appear counterintuitive at face value. It might be speculatively explainable if considered a proxy for treatment resistance, although the studies did not specifically target treatment refractoriness. Further work to test this intervention in refractory major depression would be of interest considering that poor response to treatment and polypharmacy are far from unusual.

It is also of interest that most participants in these studies were women (91%), which limits the generalisability of this intervention to men. This could be partially attributable to the epidemiology of depression, although there may be other reasons, including the possibility that women may be more agreeable to this intervention, irrespective of purely cosmetic interests or personality traits. Further studies with a much larger contribution of male patients are advisable.

In terms of limitations, it is important to mention that although Egger’s test excluded the chance of publication bias in the analyses, the number of studies included in this work is small. Hence, it is not possible to exclude completely the selective inclusion of positive studies at the expense of negative ones. The largest and more recent study by Brin et al. (2020) contributed to 56% of the sample of this meta-analysis. All the analyses were characterised by a high level of heterogeneity, which we systematically explored with meta-regressions. Some of the confounders identified could explain the level of heterogeneity. Nevertheless, there may be other factors either not measurable in individual studies or intrinsic to the heterogeneity of depressive illness that we could not take into consideration.

In conclusion, this PROSPERO registered meta-analysis is the largest and most up-to-date meta-analysis evaluating the efficacy of onabotulinumtoxinA in double-blind RCTs in major depression. The work offers a state-of-art methodology, which employs the best evidence-based approach (PRISMA), and a comprehensive qualitative assessment. Furthermore, the work evaluates in detail the risk of bias and, for the first time, systematically explores the significant heterogeneity of the results. Finally, we provide elaborate sub meta-analyses to help dissect the influence of placebo effects particularly important in the context of an intervention intrinsically open to such bias. Results indicate that onabotulinumtoxinA is more effective than placebo for the treatment of major depression within the 20–40 IU range. The efficacy is less pronounced in case of more complex medication regimens. Despite limitations in recruiting participants in mood disorders (Wise et al., 2016), more research is required to understand better the contribution to placebo effects and sex differences to the measured effect size and which depression subtypes are more likely to benefit from treatment with onabotulinumtoxinA.

## Supplemental Material

sj-docx-1-jop-10.1177_0269881121991827 – Supplemental material for Efficacy of onabotulinumtoxinA in the treatment of unipolar major depression: Systematic review, meta-analysis and meta-regression analyses of double-blind randomised controlled trials Click here for additional data file. Supplemental material, sj-docx-1-jop-10.1177_0269881121991827 for Efficacy of onabotulinumtoxinA in the treatment of unipolar major depression: Systematic review, meta-analysis and meta-regression analyses of double-blind randomised controlled trials by Danilo Arnone, Hassan Galadari, Carl J Rodgers, Linda Östlundh, Karim Abdel Aziz, Emmanuel Stip and Allan H Young in Journal of Psychopharmacology
