# Supplementary material for: Efficacy of onabotulinumtoxinA in the treatment of unipolar major depression: Systematic review, meta-analysis and meta-regression analyses of double-blind randomised controlled trials
Source: J Psychopharmacol. 2021 Mar 15;35(8):910–8. doi: 10.1177/0269881121991827 (PMC8366169; doi:10.1177/0269881121991827)
Supplement: sj-docx-1-jop-10.1177_0269881121991827 – Supplemental material for Efficacy of onabotulinumtoxinA in the treatment of unipolar major depression: Systematic review, meta-analysis and meta-regression analyses of double-blind randomised controlled trials [file sj-docx-1-jop-10.1177_0269881121991827.docx]

**Supplementary material 1**

**Literature search**

Academic databases

| **Source and search date** | **Search string** | **Results and notes** |
| --- | --- | --- |
| **PubMed**  **Coverage:**  1809-  **Search date:**  2020-11-18 | ((("Randomized Controlled Trial" [Publication Type] OR "RCT"[Title/Abstract] OR “double blind*”[Title/Abstract] OR “single blind*”[Title/Abstract] OR placebo[Title/Abstract] OR “controlled clinical trial*”[Title/Abstract] OR randomize*[Title/Abstract] OR randomis*[Title/Abstract])) AND ("Bipolar and Related Disorders"[Mesh] OR "Mood Disorders"[Mesh] OR "Depression"[Mesh] OR bipolar*[Title/Abstract] OR "affective disorder*"[Title/Abstract] OR depression[Title/Abstract] OR depressions[Title/Abstract] OR depressed[Title/Abstract] OR depressive[Title/Abstract] OR "mood disorder*"[Title/Abstract])) AND ("Botulinum Toxins"[Mesh] OR "Botulinum Toxins, Type A"[Mesh] OR "Botulinum toxin*"[Title/Abstract] OR “Clostridium botulinum A Toxin”[Title/Abstract] OR Botox[Title/Abstract] OR “OnabotulinumtoxinA”[Title/Abstract] OR “Onabotulinumtoxin A”[Title/Abstract] OR "Botulinum neurotoxin type-A"[Title/Abstract] OR “BoNT-A"[Title/Abstract] OR “Onabotulinum toxinA”[Title/Abstract] OR “Onabotulinum toxin A”[Title/Abstract])) | **Result:**  48  **Notes:**  All search terms are searched in “title” and “abstract” (here marked with TI/AB) and in MeSH (when available).  **Filters and limitations:**  No filters or limitations applied. |
| **Embase**  **Coverage:**  1947-  **Source**: Embase only  **Search date:**  2020-11-18 | ((('botulinum toxin'/de OR 'botulinum toxin a'/de OR 'botulinum toxins':ab,ti OR 'botulinum toxin':ab,ti OR 'clostridium botulinum a toxin':ab,ti OR botox:ab,ti OR 'onabotulinumtoxina':ab,ti OR 'onabotulinumtoxin a':ab,ti OR 'botulinum neurotoxin type-a':ab,ti OR 'bont-a':ab,ti OR 'onabotulinum toxina':ab,ti OR 'onabotulinum toxin a':ab,ti) AND ('mood disorder'/exp OR 'depression'/exp OR 'bipolar disorder'/exp OR bipolar*:ab,ti OR 'affective disorder*':ab,ti OR depression:ab,ti OR depressions:ab,ti OR depressed:ab,ti OR depressive:ab,ti OR 'mood disorder*':ab,ti) AND ('randomized controlled trial'/exp OR 'rct':ab,ti OR 'double blind*':ab,ti OR 'single blind*':ab,ti OR placebo:ab,ti OR 'controlled clinical trial*':ab,ti OR randomize*:ab,ti OR randomis*:ab,ti))) | **Result:**  88  **Notes:**  All search terms are searched in the fields: “title” and “abstract” (here marked with “:ab,ti”) and in the “thesaurus” (here marked with “/de”) when available.  No filters or limitations applied.  **Thesaurus (Emtree) variations compared to PubMed’s MeSH:**  “Bipolar and Related Disorders" is referred to Bipolar Disorder”. Included. |
| **Scopus**  **Coverage:**  1788-  **Search date:**  2020-11-18 | ((TI-ABS ("RCT" OR "double blind*" OR "single blind*" OR placebo OR "controlled clinical trial*" OR  randomize*  OR  randomis* ) AND (TI-ABS( "Botulinum toxin*"  OR "Clostridium botulinum A toxin" OR Botox OR "OnabotulinumtoxinA" OR "Onabotulinumtoxin A" OR  "Botulinum neurotoxin type-A" OR "BoNT-A" OR  "Onabotulinum toxinA" OR "Onabotulinum toxin A" ) AND (TITLE-ABS-KEY (bipolar* OR "affective disorders" OR “affective disorder" OR depression OR  depressions OR depressed OR depressive OR "mood disorders" OR "mood disorder")) | **Result:**  88  **Notes:**  All search terms are searched in the fields: “title”and “abstract (here marked with “TITLE-ABS-), alternative with “title”, “abstract and “keywords” (here marked with “TITLE-ABS-KEY”).  No thesaurus available.  No filters or limitations applied. |
| **APA PsycInfo**  (EBSCO)  **Coverage:**  1632-  **Search date:**  2020-11-18 | (((TI ( "RCT" OR “double blind*” OR “single blind*” OR placebo OR “controlled clinical trial*” OR randomize* OR randomis*) OR AB ( "RCT" OR “double blind*” OR “single blind*” OR placebo OR “controlled clinical trial*” OR randomize* OR randomis* ) OR  DE "Randomized Controlled Trials" OR DE "Randomized Clinical Trials" OR DE "Placebo") AND  (TI ( bipolar* OR "affective disorders" OR "affective disorder" OR depression OR depressions OR depressed OR depressive OR “mood disorder*") OR AB ( bipolar* OR "affective disorder*" OR depression OR depressions OR depressed OR depressive OR "mood disorder*" ) OR DE ("Bipolar Disorder" OR DE "Affective Disorders" OR DE "Major Depression") AND (TI ( "Botulinum toxins" OR "Botulinum toxin" OR “Clostridium botulinum A Toxin” OR Botox OR “OnabotulinumtoxinA” OR “Onabotulinumtoxin A” OR "Botulinum neurotoxin type-A" OR “BoNT-A" OR “Onabotulinum toxinA” OR “Onabotulinum toxin A” ) OR AB ( "Botulinum toxins" OR "Botulinum toxin" OR “Clostridium botulinum A Toxin” OR Botox OR “OnabotulinumtoxinA” OR “Onabotulinumtoxin A” OR "Botulinum neurotoxin type-A" OR “BoNT-A" OR “Onabotulinum toxinA” OR “Onabotulinum toxin A” )  OR DE ("Botulinum Toxin"))) | **Result:**  21  **Notes:**  All search terms are searched in the fields: “title” and “abstract” (here marked with “TI and “AB”) and in the “thesaurus” (here marked with “DE”) when available.  No filters or limitations applied.  **Thesaurus variations compared to PubMed’s MeSH:**  “Mood Disorder” is referred to “Affective Disorder “. Included  “Depression” refers to “Depression (Emotions)”.  Not included. Replaced with  “Major Depression” |
| **Web of Science**  (Core Collection)  **Coverage:**  1955-  **Search date:**  2020-11-18 | (((TOPIC: "Botulinum toxin*" OR “Clostridium botulinum A Toxin” OR Botox OR “OnabotulinumtoxinA” OR “Onabotulinumtoxin A” OR "Botulinum neurotoxin type-A" OR “BoNT-A" OR “Onabotulinum toxinA” OR “Onabotulinum toxin A”) AND (TOPIC: bipolar* OR "affective disorder*" OR depression OR depressions OR depressed OR depressive OR "mood disorder*") AND (TOPIC: "RCT" OR “double blind*”OR “single blind*” OR placebo OR “controlled clinical trial*” OR randomize* OR randomis*))) | **Result:**  185  **Notes:**  All search terms are searched in the field: “topic” (including title, abstract and author supplied keywords, here marked with “TOPIC”).  No filters or limitations applied.  No thesaurus available. |
| **Cochrane**  **Coverage:**  All years included  **Search date:**  2020-11-18 | ((("Randomized Controlled Trial" [Mesh] OR "RCT"[ti,ab,kw] OR “double blind”[ti,ab,kw] OR “double blinded”[ti,ab,kw] OR “single blind”[ti,ab,kw] OR “single blinded”[ti,ab,kw] OR placebo[ti,ab,kw] OR “controlled clinical trial”[ti,ab,kw] OR randomize*[ti,ab,kw] OR randomis*[ti,ab,kw])) AND ("Bipolar and Related Disorders"[Mesh/EXP] OR "Mood Disorders"[Mesh/EXP] OR "Depression"[Mesh/EXP] OR bipolar*[ti,ab,kw] OR "affective disorders"[ti,ab,kw] OR "affective disorder"[ti,ab,kw] OR depression[ti,ab,kw] OR depressions[ti,ab,kw] OR depressed[ti,ab,kw] OR depressive[ti,ab,kw] OR "mood disorders"[ti,ab,kw] OR "mood disorder"[ti,ab,kw])) AND ("Botulinum Toxins” [Mesh] OR "Botulinum Toxins, Type A"[Mesh] OR "Botulinum toxins"[ti,ab,kw] OR "Botulinum toxin"[ti,ab,kw] OR “Clostridium botulinum A Toxin”[ti,ab,kw] OR Botox[ti,ab,kw] OR “OnabotulinumtoxinA”[ti,ab,kw] OR “Onabotulinumtoxin A”[ti,ab,kw] OR "Botulinum neurotoxin type-A"[ti,ab,kw] OR BoNT-A[ti,ab,kw] OR “Onabotulinum toxinA”[ti,ab,kw] OR “Onabotulinum toxin A”[ti,ab,kw]))) | **Result:**  89  **Notes:**  All search terms are searched in the fields “title”, “abstract” and “keywords” (here marked with “ti,ab,kw”) and in “MeSH” when available.  **Filters and limitations:**  A filter for clinical trials is applied  **MeSH variations compared to PubMed:** None. |
| **Total number of references** | | **519** |
| **Total number of references after de-duplication** | | **347** |

**Grey sources**

| **Source** | **Search string** | **Result and notes** |
| --- | --- | --- |
| **Open Grey**  **(**<http://www.opengrey.eu/>)  **Search date:**  2020-05-01 | ((botulinum OR Botox) AND {depression OR depressive OR depressed)) | **Result:**  0  **Notes:**  Only limited search functions available. |
| **NY Academy of Medicine- Grey Literature Report**  **(**<https://catalog.nyam.org/>)  **Search date:**  2020-05-01 | ((botulinum OR Botox) AND {depression OR depressive OR depressed)) | **Result:**  0  **Notes:**  Only limited search functions available. |
| **Bielefeld Academic Search Engine** (BASE)  (<https://www.base-search.net/Search/Advanced>)  **Search date:**  2020-05-01 | tit:botulinum AND tit:depression doctype:(11* 12* 13 14 18*) | **Result:**  11  **Notes:**  Only limited search functions available.  Document types included: “book”, “book part”, “journal/newspaper”, “report” and “theses” |
| **Clinical trials.gov**  (<https://clinicaltrials.gov/>)  **Search date:**  2020-05-01 | ((botulinum OR Botox) AND {depression OR depressive OR depressed)) | **Result:**  4  **Notes:**  Only limited search functions available. |
| **Directory of Open Access Journals** (DOAJ)  (<https://doaj.org/>)  **Search date:**  2020-05-01 | (botulinum AND depression) | **Result:**  3  **Notes:**  Only limited search functions available. |
| **Total no. of references identified:** | | **18** |
| **Total no. of references after de-duplication within the grey sources and the result from the database search:** | | **11** |
